# Editorial: Women in terrestrial microbiology: 2022

**DOI:** 10.3389/fmicb.2023.1326145

**Published:** 2023-11-27

**Authors:** Paola Grenni, Katharina Kujala, Anna Barra Caracciolo

**Affiliations:** ^1^Water Research Institute of the National Research Council, Montelibretti, Italy; ^2^National Biodiversity Future Center (NBFC), Palermo, Italy; ^3^Water, Energy, and Environmental Engineering Research Unit, University of Oulu, Oulu, Finland

**Keywords:** PGPB, bioplastic, hyperarid soil microbiome, fungal decomposition, sustainable horticultural substrates, biodeteriogens in Egyptian pyramids, soil rare bacteria

Although we have been discussing gender equality for many decades, in 2023 we have not yet reached it. When looking at science, and especially STEM (science, technology, engineering, and mathematics) research, it is clear that more efforts are needed to achieve gender equality. Women are still underrepresented in STEM research fields, with only 30% of the world's researchers being women, according to the UNESCO Institute for Statistics. Higher dropout rates, resulting in shorter academic careers, are common for women (Huang et al., 2020). While the share of female authors in scientific publications has increased over the last few decades (Huang et al., 2020; Sarabi and Smith, 2023), male authors still dominate the publishing landscape, and even when male and female authors have contributed equally to a publication (i.e., shared first authorship), it is more common to see the male author's name mentioned before the female author's (Broderick and Casadevall, 2019). Women are overall less likely to receive credit for their work than their male counterparts, as male researchers more often receive co-authorship than female researchers for similar tasks (Ross et al., 2022). This brings on a vicious cycle of lower visibility, lower impact, and, in consequence, lower funding and career opportunities (Van den Besselaar and Sandström, 2017). In particular, the higher career ranks, such as full professors, are still very much male-dominated (Van den Besselaar and Sandström, 2017).

There is evidence that gender-diverse teams tend to produce research output with a higher degree of novelty and impact (Sarabi and Smith, 2023). While the reasons for this are still unclear, it is likely that the integration and empowerment of women (i.e., acknowledgment and incorporation of their expertise) rather than strict gender ratios are the keys to high-impact research (Love et al., 2022). It is therefore in the interest of science and society to promote gender equality in STEM fields. To leverage female authors in STEM fields and give them a platform to promote their findings, Frontiers has launched a series of Research Topics celebrating International Women's Day. All publications on these Research Topics have a female researcher as the first author and, for some papers, also as the corresponding author. Our Research Topic, Women in Terrestrial Microbiology: 2022, features eight articles covering a wide range of terrestrial microbiology topics in which female researchers played the main role.

The articles presented discuss microbes from extreme habitats (Aguilera-Torres et al.; Demergasso et al.), microbes beneficial to plants (Boak et al.; Cuartero et al.; Pot et al.), microbes involved in organic matter degradation (Brabcová et al.; Bandini et al.), and microbial threats to ancient monuments (Rizk et al.).

Extreme environments such as the arid Atacama Desert or the exposed slopes of the Andes challenge microbial communities with various stressors such as desiccation, large temperature fluctuations, and changes in moisture. Demergasso et al. demonstrated in a simulated rainfall event that bacterial communities in the Atacama Desert have the potential to respond quickly to the improved conditions and initiate growth. In the Andes, plant growth-promoting bacteria were shown by Aguilera-Torres et al. to alleviate the environmental stress on xerophytic plants. Plant-beneficial bacteria were also the focus of Boak et al., who found that the type-II secretion systems of *Pseudomonas chloroaphis* are a valuable weapon against competitors and predators, thus helping to shape the microbial communities of the rhizosphere. Cuartero et al., on the other hand, investigated the impact of the sustainable farming practice of intercropping on soil microbial diversity and soil C and N cycling. Their study demonstrated the beneficial effects of intercropping on both microbial diversity and the abundance of plant-beneficial microbes. More sustainable practices were also the inspiration for the study by Pot et al., who looked into materials to replace peat, a popular growing substrate in horticulture, with more sustainable alternatives. They found that many of the tested options, e.g., green or vegetable composts, showed higher numbers of potentially plant-beneficial microbes than the traditional peat-based substrates. Microbial communities involved in organic matter degradation were the focus of the study by Brabcová et al.. They showed how fungal communities in decaying deadwood change over the course of the decay process. Moreover, these changes can be linked to microclimate factors such as pH and temperature in the wood. On a similar topic, but from a more applied perspective, Bandini et al. focused on how bioplastics, which are widely advocated as sustainable alternatives to traditional plastics, affect microbial communities during anaerobic digestion and aerobic composting. Indeed, the authors found a pronounced impact, which may indicate a need to adjust process parameters for digestion and composting if bioplastics are to be degraded in these systems in the future. The last study in our Research Topic dedicated to women researchers in Terrestrial microbiology takes us to Egypt, where Rizk et al. set out to characterize potential microbial threats to the pyramids. They found that the microbial communities in and on the stones of the pyramids also comprise such microbes that could increase the risk of physical and chemical deterioration of these valuable cultural heritage monuments, which needs to be considered in conservation efforts.

This Research Topic is a valid example of how women are able to coordinate high-level research.

## Author contributions

PG: Writing—original draft, Writing—review & editing. KK: Writing—original draft, Writing—review & editing. ABC: Writing—review & editing.
